# Supraorbital eyebrow approach for ligation of an anterior fossa combined dural and pial arteriovenous fistula

**DOI:** 10.3171/2025.7.FOCVID2559

**Published:** 2025-10-01

**Authors:** Ehsan Dowlati, Justin Turpin, Daniel Toscano, Kevin Shah, Amir R. Dehdashti

**Affiliations:** Department of Neurosurgery, Northwell Health/Donald and Barbara Zucker School of Medicine at Hofstra, Manhasset, New York

**Keywords:** anterior fossa, arteriovenous fistula, clip ligation, eyebrow, supraorbital, pial fistula

## Abstract

Microsurgical treatment of arteriovenous fistulas (AVFs) of the anterior fossa is indicated when endovascular approaches carry excessive risk due to the involvement of the ethmoidal arteries. A 72-year-old male presenting with episodes of blurred vision was found to have an atypical Cognard type IV ethmoidal AVF with combined dural and pial supply from an orbitofrontal branch. He underwent supraorbital craniotomy via eyebrow incision for successful clip ligation of the AVF at the cribriform plate. The supraorbital eyebrow approach offers a minimally invasive corridor, enabling direct visualization and disconnection of anterior fossa AVFs while minimizing brain retraction and cosmetic impact.

The video can be found here: https://stream.cadmore.media/r10.3171/2025.7.FOCVID2559

## Transcript

In this video, we present the case of a supraorbital craniotomy via eyebrow incision for clip ligation of an anterior fossa combined dural and pial arteriovenous fistula (AVF).

### 0:31 Patient History and Exam.

Patient is a 72-year-old male who presents to medical attention with episodes of blurred vision. He has no contributory past medical or surgical history and is otherwise neurologically intact. He was seen by ophthalmology and referred for an MRI shown here, which demonstrated concerns for an anterior fossa vascular lesion.

### 0:51 Preoperative Imaging.

Further imaging workup with an MRA shown here demonstrated that there is abnormal vasculature anterior to the right frontal lobe and anterior fossa, consistent with a shunting vascular lesion such as an AVF. He underwent diagnostic angiogram, which demonstrated an ethmoidal Borden type III/Cognard type IV dural AVF with additional pial supply from a branch of the right orbitofrontal artery. The fistulous point is at the cribriform plate and translates into a dilated anterior ectatic cortical vein that goes superiorly and toward the superior sagittal sinus. Views of the right ICA injection shown here demonstrate the complex angioarchitecture of the AVF with dural and pial supply. There is no direct supply from the ECA or left ICA. Three-dimensional reconstructions from the Dyna CT better demonstrate the fistulous point of the lesion.

### 1:51 Treatment Options.

Dural AVFs can have varied clinical presentation based on location.^1^ The patient’s presenting visual symptoms can be attributed to a vascular steal phenomenon. Given the high-grade classification of the fistula involving direct drainage into a cortical vein with ectasia, treatment was indicated. Dural arteriovenous fistulas with cortical venous reflux have a higher risk of hemorrhage (over 8% annual risk).^2,3^ We proposed treatment via a right supraorbital craniotomy via eyebrow incision for clip ligation of the arteriovenous fistula with intraoperative angiography.

Alternative treatments were discussed with the patient including: Observation, which is not appropriate given high annual risk of hemorrhage. Endovascular embolization was deemed too high-risk given ethmoidal arteries arise from the ophthalmic artery and there is risk of visual deficit.^4^ Radiosurgery is associated with significant delay in time to cure during which he may be at risk of hemorrhage and this should be reserved for patients who are not good surgical candidates.^5^ Other surgical approaches including bicoronal craniotomy or pterional craniotomy for clip ligation were entertained;^6^ however, these approaches require larger incisions and craniotomies and the patient opted for a more minimally invasive approach due to his receding hairline.

### 3:08 Positioning and Equipment.

For positioning, the patient is supine with the head fixed in a radiolucent Mayfield holder rotated 30° toward the left side in slight extension.

Necessary equipment includes surgical microscope, neuromonitoring, neuronavigation for craniotomy planning and approach trajectory, high-speed drill, aneurysm clips, indocyanine green, and equipment needed for a transfemoral diagnostic intraoperative angiogram

The incision is shown here running just above the eyebrow with a supraorbital notch marking the medial extent and frontal root of zygoma the lateral extent.

### 3:42 Supraorbital Approach.

After incision is made, the periosteum is dissected off of the frontal bone down to the orbital rim and the temporalis is dissected off and reflected laterally. Navigation is used to mark the lateral extent of the frontal sinus.

A burr hole is made at the keyhole, and after peeling away the dura the craniotomy is extended with a router drill bit and the bone flap is removed. Further drilling of the anterior fossa floor and orbital roof is performed to create a flat access plane for our approach.

### 4:16 Intradural Exposure.

The dura is opened and reflected anteriorly against the orbital rim and the microscope is brought in. The anterior fossa floor is followed. CSF is gradually released from cisterns for brain relaxation. The draining vein is apparent along the anterior surface of the right frontal lobe. For better visualization, further medial drilling is performed of the medial frontal bone.

### 4:56 Identification and Dissection of the Fistula.

The draining vein is identified and followed inferomedially toward the cribriform plate at the anterior fossa floor. The fistulous point is dissected and isolated. Indocyanine green video angiography is performed for a baseline demonstrating arterialized draining vein.

Next, a clip is placed as proximal as possible at the skull base. Micro-Doppler assessment demonstrates persistent pulsatile flow within the draining vein. This is due to the pial supply from the orbitofrontal branch, which is more distal.

Next, our attention is turned to this orbitofrontal artery with a branch coursing along the anteroinferior surface of the frontal lobe. We circumferentially dissect around this feeding artery and follow its course toward the fistulous point and identify the connection of this branch to the draining vein. Isolating this pial arterial feeder allows us a complete view of the fistulous point with the two feeding arteries and arterialized draining vein. Once isolated, a second clip is placed on this feeding artery as close as possible to the draining vein. We confirm with micro-Doppler that the draining vein is no longer pulsatile. We proceed with coagulation and disconnection of the fistula just distal to the feeding arteries.

### 6:42 Intraoperative Angiogram and Closure.

Next, we remove the surgical microscope from the field and prepare for a transfemoral intraoperative angiogram, which is performed in our hybrid operating suite. AP and oblique views of a right ICA injection are shown here demonstrating obliteration of the dAVF and no early venous shunting.

Once obliteration of the fistula is confirmed, we turn our attention to closure. We note a very small breach of the frontal sinus, which we repair using bone wax, a muscle patch, and fibrin sealant. The dura is reapproximated and reinforced with a synthetic dural graft. The craniotomy flap is secured with titanium plates and screws and any gaps are covered with polymethyl methacrylate cement. Closure of the wound is performed after hemostasis and copious irrigation. Skin closure is done with subcuticular monofilament suture.

### 7:28 Postoperative Imaging and Course.

The patient tolerated procedure well and remained neurologically intact. He was monitored for signs of orbital hematoma in the ICU overnight. On postoperative day 1, CT head demonstrated expected changes with no signs of new hemorrhage or ischemia. He was discharged home on postoperative day 2. At 2-week and 3-month follow-up, he remains neurologically intact with no further episodes of visual disturbance. Six-month follow-up MRI and MRA demonstrate complete resolution of the AVF and no concern for any shunting lesion.

### 8:03 Conclusions.

We present a complex case of a combined dural and pial AVF managed successfully with microsurgical ligation. Although this can be performed via different approaches, the supraorbital eyebrow approach offers a minimally invasive corridor, enabling direct visualization and disconnection of anterior fossa AVFs while minimizing brain retraction and cosmetic impact.^7^ In rare cases, as in our case, dAVFs can have combined pial contribution. It is important to recognize this in surgical planning to achieve complete cure of the fistula.

## Disclosures

The authors report no conflict of interest concerning the materials or methods used in this study or the findings specified in this publication.

## Author Contributions

Primary surgeon: Shah, Dehdashti. Assistant surgeon: Dowlati, Toscano. Editing and drafting the video and abstract: Dowlati, Turpin. Critically revising the work: Dowlati, Shah. Reviewed submitted version of the work: Dowlati. Approved the final version of the work on behalf of all authors: Dowlati.

## Supplemental Information

### Patient Informed Consent

The necessary patient informed consent was obtained in this study.
